# Destabilization of Surfactant-Dispersed Carbon Nanotubes by Anions

**DOI:** 10.1186/s11671-017-1850-1

**Published:** 2017-01-31

**Authors:** Atsushi Hirano, Weilu Gao, Xiaowei He, Junichiro Kono

**Affiliations:** 1 Nanomaterials Research Institute, National Institute of Advanced Industrial Science and Technology (AIST), Tsukuba, Ibaraki 305-8565 Japan; 2 Department of Electrical and Computer Engineering, Rice University, Houston, TX 77005 USA; 3 Department of Physics and Astronomy, Rice University, Houston, TX 77005 USA; 4 Department of Materials Science and NanoEngineering, Rice University, Houston, TX 77005 USA

**Keywords:** Carbon nanotube, Colloidal stability, Separation, Surfactant, Anion

## Abstract

The colloidal stability of surfactant-dispersed single-wall carbon nanotubes (SWCNTs) is determined by microscopic physicochemical processes, such as association, partitioning, and adsorption propensities. These processes can be controlled by the addition of solutes. While the effects of cations on the colloidal stability of SWCNTs are relatively well understood, little is known about the effects of anions. In this study, we examined the effects of anions on the stability of SWCNTs dispersed by sodium dodecyl sulfate (SDS) using sodium salts, such as NaCl and NaSCN. We observed that the intensity of the radial breathing mode Raman peaks rapidly decreased as the salts were added, even at concentrations less than 25 mM, indicating the association of SWCNTs. The effect was stronger with NaSCN than NaCl. We propose that the association of SWCNTs was caused by thermodynamic destabilization of SDS assemblies on SWCNT surfaces by these salts, which was confirmed through SWCNT separation experiments using aqueous two-phase extraction and gel chromatography. These results demonstrate that neutral salts can be used to control the colloidal stability of surfactant-dispersed SWCNTs.

**Electronic supplementary material:**

The online version of this article (doi:10.1186/s11671-017-1850-1) contains supplementary material, which is available to authorized users.

## Background

Single-wall carbon nanotubes (SWCNTs) are unique one-dimensional materials with promising properties for use in nanotechnology applications, including photonics, electronics, and drug delivery [1–4]. For basic research purposes, SWCNTs are often prepared in aqueous solution with the assistance of surfactant because they themselves are insoluble [5–7]. The physical and physicochemical properties of dispersed SWCNTs are significantly affected by solutes, including neutral salts, chaotropes, organic compounds, and coexisting surfactants; in particular, the optical and colloidal properties are readily altered by solutes [8–18]. These solute effects have been extensively investigated, especially for SWCNTs dispersed with sodium dodecyl sulfate (SDS), a negatively charged surfactant.

Previous studies on the effects of neutral salts on dispersed SWCNTs have primarily examined the role of cations [8, 9, 11, 12, 18]. For example, the photoluminescence (PL) intensity of SDS-dispersed SWCNTs was increased with the addition of NaCl at a concentration less than 50 mM; in contrast, the PL intensity decreased when the concentration of NaCl exceeded 100 mM, although the tendency depended on the chirality. The effect observed at low concentrations was more significant for higher-valent cations (i.e., Na^+^ > Mg^2+^ > Er^3+^), which can be explained in terms of the screening of electrostatic forces [8]. Intertube repulsion is enhanced by steric repulsive forces owing to increased SDS packing density caused by the screening effect at low ion concentrations, whereas intertube attraction is enhanced by van der Waals forces dominating over the steric repulsive forces at high ion concentrations. Similar phenomena were observed in the comparison of PL intensities in the presence of different-size cations (i.e., Na^+^ and Cs^+^) [11], which can also be explained by the screening effect of cations on SDS headgroups [18]. In fact, the critical micelle concentration of SDS depends on the nature of the cation [19]. However, in contrast to these reports, little is known about the effects of anions on the properties of surfactant-dispersed SWCNTs.

Anions affect the hydrophobic interactions between hydrophobic solutes in aqueous solutions [20]. The anionic effects depend on the nature of the anion. Their effectiveness is typically in the following order: SO_4_^2−^ > F^−^ > Cl^−^ > I^−^ > SCN^−^. This order is generally called the Hofmeister series (or the lyotropic series). The anions toward the left in the series, such as SO_4_^2−^, are more effective for enhancing hydrophobic interactions and are called kosmotropes, whereas the anions toward the right, such as SCN^−^, are more effective for reducing hydrophobic interactions and are called chaotropes. This alteration of interactions by the salts in the Hofmeister series affects the thermodynamic stability of the solutes, including organic solute solubility, protein folding, and DNA melting [20–23]. The mechanism of the effect is partly interpreted in terms of surface tension or hydrogen bonding of the solutions and direct interactions with the solutes [20, 24–27]. In addition to neutral salts, urea also induces a chaotropic effect [28]. In contrast to cations, the critical micelle concentrations of positively charged surfactants depend on the nature of the anion [19].

Interactions between chaotropes and SWCNTs have been investigated in various systems through experiments and simulations. For example, a previous study by Hirano et al. showed that SWCNTs dispersed by SDS are eluted from hydrogel columns by urea at several molar [12]. In addition, the dispersibility of SWCNTs with protein is improved by urea at several molar [13]. Molecular dynamics simulations of a SWCNT in a urea solution showed preferential binding to the SWCNT sidewall at 7 M [29]. NaSCN at several hundred millimolar was reported to elute DNA-dispersed SWCNTs from an ion-exchange column [30]. These results suggest that SWCNTs are stabilized in aqueous media in the presence of chaotropes at high concentrations, typically greater than 1 M. In addition to the interaction of solutes with SWCNTs, some neutral salts can thermodynamically destabilize SDS micelles [31, 32]. Recently, NaSCN at low concentrations was found to alter the colloidal stability of SWCNTs dispersed by negatively charged surfactants in aqueous polymer systems [10, 33, 34]. However, the effect of anions has not been addressed in detail.

In this study, we investigated the colloidal stability of surfactant-dispersed SWCNTs in the presence of low concentrations (0–25 mM) of solutes, including neutral salts and urea. We characterized the effects of anions through absorption spectra, Raman spectra, the degree of partitioning in aqueous two-phase (ATP) systems, and the amount of adsorption on hydrogels [10, 12, 33, 35]. The experimental results showed that NaSCN strongly alters Raman intensities, the partitioning in ATP systems, and the adsorbability onto hydrogel surfaces, which demonstrates that thiocyanate ions thermodynamically destabilize SDS-dispersed SWCNTs; such destabilization was accounted for by the preferential exclusion of thiocyanate ions from SDS assemblies on SWCNT surfaces but not by the chaotropic effect. Importantly, previous research addressing partitioning in ATP systems has suggested that SWCNTs would be stabilized by thiocyanate ions [10]. The present approach thus revealed the anionic dependence of the colloidal stabilities of surfactant-dispersed SWCNTs. This information is useful not only for understanding the basic properties of SWCNT colloids in electrolytes but also for manipulating SWCNTs in aqueous solutions, e.g., SWCNT separation processes.

## Methods

### Chemicals

Raw SWCNTs produced by high-pressure catalytic CO (HiPco) decomposition were obtained from the HiPco Laboratory at Rice University. NaSCN, urea, potassium hexachloroiridate (IV), SDS, sodium deoxycholate (DOC), and sodium cholate (SC) were purchased from Sigma-Aldrich. Hydrochloric acid solution was purchased from EMD Millipore. NaCl and sodium hydroxide were purchased from Avantor Performance Materials. Polyethylene glycol (PEG), with a molecular weight of approximately 6000 (polyethylene glycol 6000), was purchased from Alfa Aesar, and dextran, with a molecular weight of approximately 70,000 (Dextran 70), was from TCI America.

### Preparation of the SWCNT Dispersion

Raw SWCNTs were used as the starting material. SDS was used as a dispersant for the SWCNTs. Ten milligrams of HiPco SWCNTs was dispersed with 20 mL of a 1 wt% SDS solution using an ultrasonic homogenizer (XL-2000, Sonicator, Qsonica, LLC) with a 1/4-inch probe for 1 h at 30 W. To prevent heating during ultrasonication, the bottle containing the sample was immersed in an ice bath. The dispersed sample was centrifuged at 210,000×*g* for 1 h using an ultracentrifuge (Optima L-80XP Ultracentrifuge, Beckman Coulter) to remove the residual catalytic metal particles, nanotube bundles, and other impurities. The upper 70% of the supernatant was collected as a debundled SWCNT dispersion. The SWCNTs dispersed in each surfactant solution were diluted to an absorbance (1-mm path length) of 1 at 220 nm using 1 wt% SDS.

### Measurement of the Absorption Spectra and Raman Spectra

The absorption spectra of the SWCNTs were recorded using a UV-vis-NIR scanning spectrophotometer (UV-3101PC, Shimadzu) equipped with a quartz cell with a path length of 1 or 10 mm. Raman spectra for the radial breathing mode (RBM) of the SWCNTs were collected using an inVia Raman Microscope (Renishaw) equipped with a 785-nm laser. The Raman spectra of the samples were recorded at excitation wavelengths of 785 nm. The data for water were subtracted from the raw data for each SWCNT solution; note that the solutes and surfactants themselves insignificantly affected the spectra in RBM mode.

### SWCNT Partitioning Experiment in Aqueous Two-Phase Systems

Partitioning experiments on the SWCNTs in the ATP system were conducted by mixing the debundled SWCNT dispersion with polymers and surfactants to produce a mixture containing 6 wt% PEG, 6 wt% dextran, 0.4 wt% SDS, and 0.9 wt% SC, with or without 25 mM solutes (i.e., NaCl, NaSCN, and urea). Thirty minutes after mixing, the mixture was gently centrifuged to obtain the two-phase separations.

### SWCNT Adsorption Experiment in Hydrogel Columns

Hydrogel columns containing Sephacryl (Sephacryl S200 HR, GE Healthcare) were used to examine the adsorbability of SWCNTs onto hydrogel surfaces. The debundled SWCNT dispersions (0.3 mL in 1 wt% SDS) were loaded onto the top of a column that contained approximately 0.6 mL of Sephacryl equilibrated with 1 wt% SDS. A 1-mL aliquot of a 1 wt% SDS solution was added to the columns to obtain flow-through fractions. Subsequent fractions were eluted with 1 mL of 1 wt% SDS solution with or without 25 mM solute or with 1 wt% DOC. Finally, the remaining material was eluted with 1 wt% DOC, which resulted in complete elution.

### Measurement of the Photoluminescence Spectra

The excitation light was generated with a Xenon lamp and a monochromator (MicroHR, Horiba). A cold mirror and a series of optical filters were used to separate the excitation and emission light. The emission light was collected by a lens and was analyzed with a spectrometer (Acton SpectraPro 2300i, Princeton Instruments) equipped with a liquid-nitrogen-cooled InGaAs one-dimensional array.

## Results and Discussion

### Characterization of SWCNTs in the Presence of Solutes

SWCNTs produced by the HiPco process were used in this study. The SWCNTs typically had a diameter distribution of 0.8–1.2 nm, which gives spectral absorption mainly at wavelengths less than approximately 1400 nm. Optical transitions for metallic SWCNTs were observed at approximately 400–620 (M_11_ band). π-Plasmon absorption of the SWCNTs was detected at approximately 220 and 280 nm because of the collective excitation of the π-electron systems polarized across and along the SWCNT axes, respectively [36]. Optical transitions for semiconducting SWCNTs were observed at approximately 940–1350 nm (S_11_ band) and 620–940 nm (S_22_ band) (Additional files 1: Figure S1). The chirality distribution of the semiconducting SWCNTs was determined using photoluminescence spectroscopy (Additional files 1: Figure S2), which indicates the presence of (6,5), (8,3), (7,5), (8,4), (10,2), (9,4), and (10,3) nanotubes in the solution.

The SWCNTs dispersed by SDS were mixed with different solute solutions. The spectral peak intensities of the SDS-dispersed SWCNTs with 25 mM NaSCN were comparable to those with no additive. NaCl, urea, and their mixture showed similar results (Additional files 1: Figure S3). Additional salts, i.e., NaBr and NaNO_3_, exhibited almost identical profiles, within experimental error (data not shown). Urea and NaSCN, which are known as chaotropes, have the ability to solubilize hydrophobic solutes in aqueous solutions [21, 28]. Such effects of chaotropes, i.e., chaotropic effects, should also be demonstrated by bare SWCNT surfaces, which are hydrophobic. In fact, favorable interactions of urea with SWCNTs have been suggested by experiments and molecular dynamics simulations [12, 13, 29]. If the chaotropic effect has appeared, urea as well as NaSCN would have altered the absorption spectra because of dissociation of the surfactant molecules from the SWCNTs. However, despite the potential ability of NaSCN and urea, a chaotropic effect on the SDS-dispersed SWCNTs was not observed at concentrations less than 25 mM, as will be discussed in the following sections.

### Raman Spectra of SWCNTs in the Presence of Solutes

**Fig. 1:**
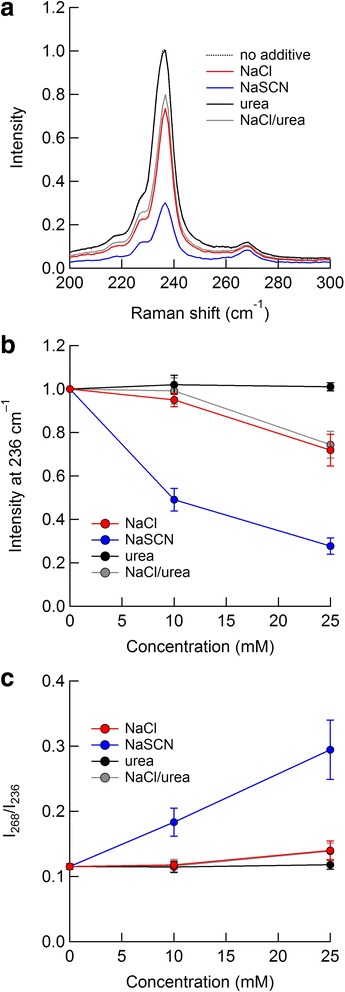
**a** Raman spectra of SWCNTs excited at 785 nm in the presence and absence of 25 mM solutes, expressed relative to the intensity at 236 cm^−1^ for the no additive sample; note that the spectra for no additive and urea overlap each other. **b** Solute concentration dependence of the Raman spectra at 236 cm^−1^. **c** Ratios of the spectral intensity at 268 cm^−1^ to that at 236 cm^−1^ as a function of the salt concentration

Because the absorption spectra of the SDS-dispersed SWCNTs in the S_11_ band were insignificantly affected by the solutes, it was concluded that the SWCNTs were not oxidized by the solutes (Additional file 1: Figure S1); note that the oxidation would potentially affect the structure of SDS on the SWCNTs [37]. However, it was possible that association of the SDS-dispersed SWCNTs was induced by the solutes [38], which may not be significantly reflected in the absorption spectra. Raman spectroscopy can be used to examine the association of SWCNTs. Intertube electrical contact broadens interband transitions and shifts them to lower energy, which can significantly alter the Raman spectra of SWCNTs through slight broadening and shifting of the interband transitions [39, 40]. Figure 1a depicts the Raman spectra of SWCNTs excited at 785 nm in the presence of 25 mM of different solutes, i.e., NaCl, NaSCN, urea, and a NaCl/urea mixture containing 25 mM NaCl and 25 mM urea, which are expressed as relative to the intensity at 236 cm^−1^ for the control sample, i.e., no additive sample. A few peaks and shoulders appeared at 200–300 cm^−1^. The position of the most intense peak was 236 cm^−1^, which corresponds to overlap of the spectra for (11,3) and (12,1) tubes [14, 40]. In addition, the small peak at 268 cm^−1^ can be assigned to (11,0) or (10,2) tubes [14, 39–41], and the other spectral shoulders at approximately 218 and 228 cm^−1^ can be assigned to (9,7) and (10,5) tubes, respectively [14, 40, 41]. The solute concentration dependence of the intensity at 236 cm^−1^ is shown in Fig. 1b. The spectral intensity showed a substantial decrease in the presence of NaSCN and slight decreases in the presence of NaCl and the NaCl/urea mixture. At 10 mM, the intensity was constant or slightly decreased for NaCl and the NaCl/urea mixture, whereas it decreased by half for NaSCN. While the effect of NaCl was comparable to that of the NaCl/urea mixture, the effect of urea was negligible, even at 25 mM, which indicates that urea has no effect on the Raman spectra at these concentrations.

Thermodynamic measurements for the solubilization of a phenyl group using two amino acids, i.e., phenylalanine and alanine (Additional files 1: Figure S4), based on the method of Nozaki and Tanford [28], showed that NaSCN and urea have similar stabilization effects on the phenyl group (Additional files 1: Table S1). Briefly, the transfer free energy of 1 M NaSCN and 1 M urea was approximately −0.4 kJ/mol for the phenyl group, whereas that of NaCl was approximately 0.3 kJ/mol. These results imply that NaSCN and urea favorably interact with the aromatic sidewalls of SWCNTs. However, despite the potential chaotropic effect of these solutes, the inactivity of urea on SWCNTs indicates a lack of favorable interaction between the chaotropes and the SWCNT sidewalls at low concentrations. In addition, there were less significant or insignificant differences in the Raman spectral profiles of NaCl, NaBr, and NaNO_3_ compared with the difference in the profiles of NaCl and NaSCN (data not shown). These results imply that the charge of the SDS-dispersed SWCNTs such as zeta potential is not practically affected by the anions at this concentration compared with cations [8], although it is difficult to detect such effects because of the large amount of the coexisting micelles.

The ratio of the intensity at 268 cm^−1^ to that at 236 cm^−1^ (*I*_268_/*I*_236_) was plotted in Fig. 1c. As expected from the spectra in Fig. 1a, the ratios dramatically increased in the presence of NaSCN. Lorentzian decomposition of the Raman spectra at 0 and 25 mM NaSCN indicated that the peak intensity at approximately 236 cm^−1^ was substantially reduced by the addition of NaSCN, whereas the intensity at approximately 268 cm^−1^ was almost retained (Additional files 1: Figure S5), which is consistent with the reported results concerning SWCNT association [39–41]. Because absorption spectra were insignificantly affected by these solutes (Additional files 1: Figure S1), the Raman spectral changes were attributed to slight alteration of the interband transitions. The observed spectral attenuation by these salts is attributable to the association of SWCNTs, which means that individual SWCNTs dispersed by SDS are destabilized by neutral salts, especially NaSCN.

### Solute Effects on SWCNT Partitioning in the Aqueous Two-Phase System

Neutral salts should have a destabilizing effect on SDS-dispersed SWCNTs. Khripin et al. reported that partitioning of the SWCNTs in ATP systems depends on the concentration and the nature of the salt in terms of thermodynamic stability [10]. We thus expected that the alteration of the stability of the SWCNTs is reflected in the partitioning in the ATP system. Here, the ATP experiments were conducted with NaCl, NaSCN, urea, and a NaCl/urea mixture to determine the effect of these solutes on the stability of SWCNTs. The ATP systems were composed of 6 wt% PEG, 6 wt% dextran, 0.4 wt% SDS and 0.9 wt% SC, similar to the ambient conditions used in a previous study [42].

**Fig. 2:**
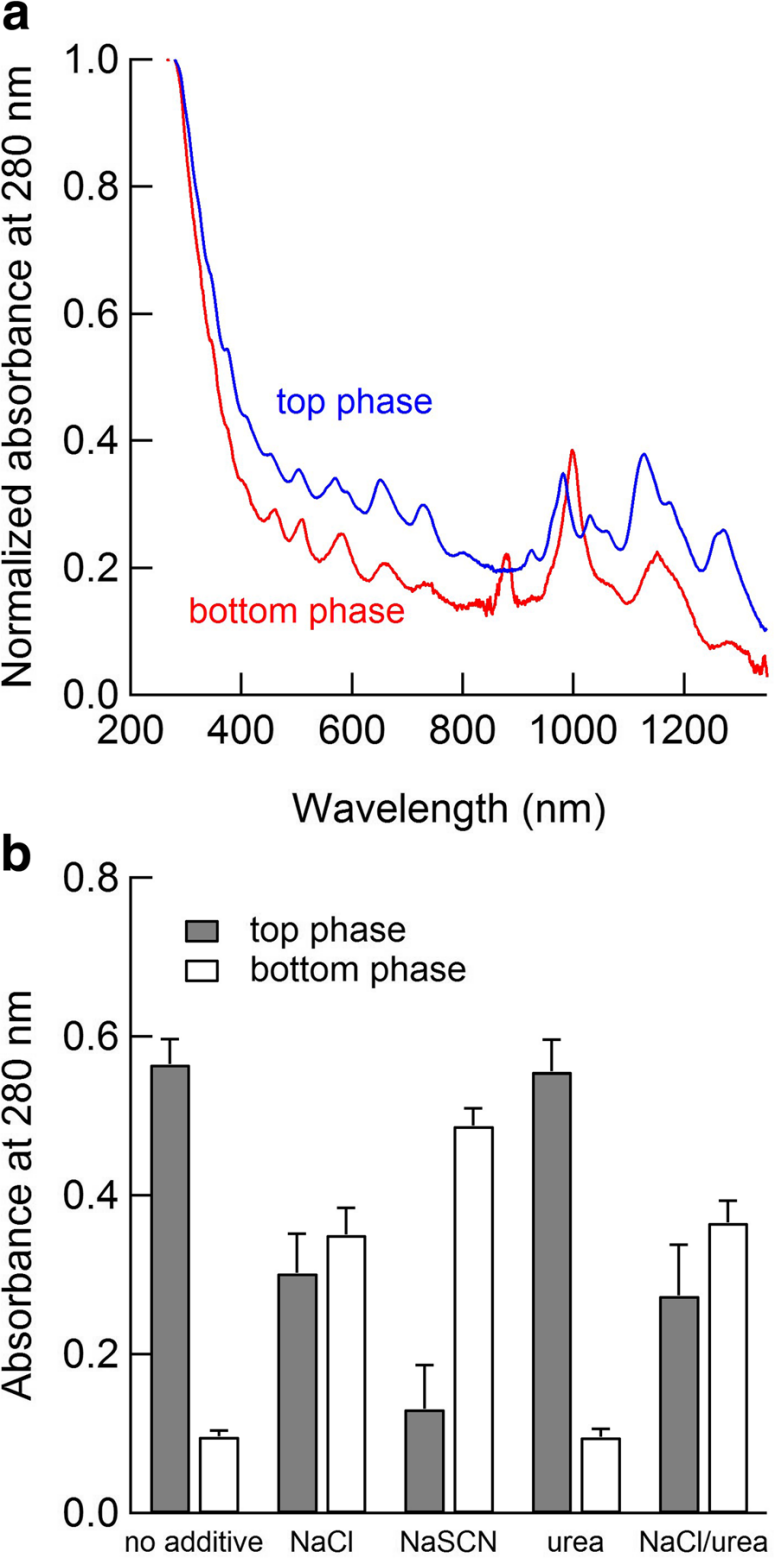
**a** Normalized absorption spectra of the SWCNTs at 280 nm for the top and bottom phases in the ATP system. **b** Absorbance of the SWCNTs at 280 nm for each phase in the presence and absence of different solutes at 25 mM

Figure 2a shows the absorption spectra of the control sample, i.e., no additive sample, normalized at 280 nm, which were obtained from the top phase after threefold dilution into 1 wt% DOC solution or from the bottom phase after the tenfold dilution. The top phase (PEG-rich phase) contains more semiconducting SWCNTs than the bottom phase (dextran-rich phase) because semiconducting SWCNTs are relatively hydrophobic [10]. As expected, the top phase had more peaks in the S_11_ and S_22_ bands than the bottom phase, whereas the bottom phase had relatively clear peaks in the M_11_ band. Figure 2b shows the absorbance values at 280 nm for each phase, with and without different solutes, which were obtained by the threefold dilution for the top phase and the tenfold dilution for the bottom phase. Most of the SWCNTs remained in the top phase in the absence of solutes, whereas NaCl pushed approximately half of the SWCNTs into the bottom phase; the results were similar for the NaCl/urea mixture. NaSCN pushed most of the SWCNTs into the bottom phase, and urea was ineffective.

The remarkable effect of NaSCN on the stability of the SWCNTs in the ATP system was examined based on the partition coefficient of NaSCN, *K* = *C*_top_/*C*_bottom_, where *C*_top_ is the concentration of NaSCN in the top phase and *C*_bottom_ is the concentration in the bottom phase. The partition coefficient of NaSCN was approximately 1.1 at 10–50 mM, indicating a higher concentration of NaSCN in the top phase than in the bottom phase (Additional files 1: Figure S6). This result supports the hypothesis that NaSCN causes destabilization of SWCNTs, namely, SWCNTs favor the phase with a lower concentration of NaSCN. Khripin et al. reported that the partitioning of SWCNTs in ATP systems was altered by the addition of 0–50 mM NaSCN [10]. They attributed the alteration of the partitioning to the chaotropic effect reducing the solvation energy, i.e., stabilization, of the SWCNTs. However, the present results indicate that the alteration of the partitioning by NaSCN was induced by destabilization of the SWCNTs. If the SWCNTs had been stabilized by NaSCN, they would have migrated to the top phase since NaSCN was more concentrated in the top phase. Although the molecular mechanism of the effect of NaSCN on the stability of SDS-dispersed SWCNTs is unclear, such destabilization by NaSCN may explain the precipitation of SWCNTs in polymer systems [34]. In addition, the fact that urea has an insignificant effect on partitioning suggests that the chaotropic effect does not occur at concentrations less than 25 mM.

### Solute Effects on SWCNT Adsorption onto Hydrogels

The effect of the solutes on the colloidal stability of SDS-dispersed SWCNTs was further examined by adsorption experiments onto hydrogel surfaces containing dextran structures. Metallic SWCNTs show weaker interactions with hydrogels, and semiconducting SWCNTs have stronger interactions, which allows separation of metal/semiconductor SWCNTs using hydrogel columns [12, 35, 43]. NaSCN interacts more favorably with dextran than with water, and the interaction difference is more pronounced than for NaCl [44, 45]. The SDS-dispersed SWCNTs should therefore be desorbed from the hydrogel surfaces owing to the relative destabilization of the SWCNTs on the hydrogel surfaces compared to those in the mobile phase. The adsorbability of the SDS-dispersed SWCNTs onto hydrogel equilibrated with 1 wt% SDS was thus examined using hydrogel columns with or without solutes. The hydrogel used here was Sephacryl S200, which is composed of allyl dextran covalently cross-linked with *N*,*N*’-methylenebisacrylamide.

**Fig. 3:**
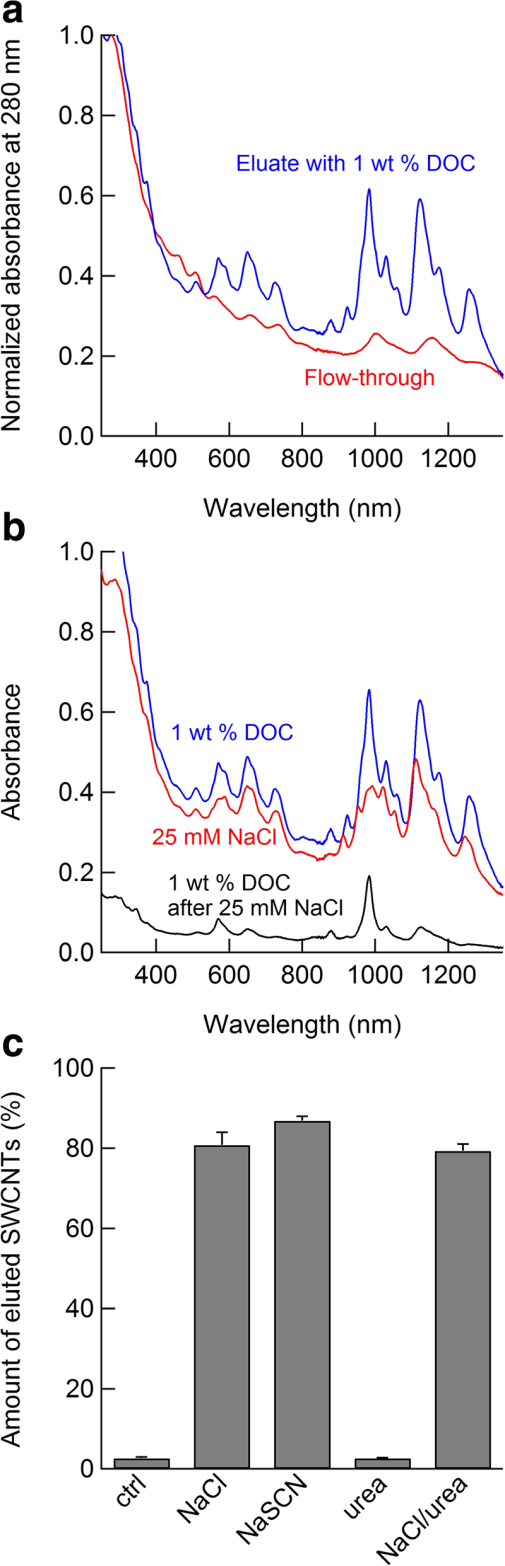
**a** Normalized absorption spectra at 280 nm for SWCNTs separated using hydrogel columns. **b** Absorption spectra of the SWCNTs eluted with a 1 wt% DOC solution, a 25 mM NaCl/1 wt% SDS mixture or a subsequent 1 wt% DOC solution. **c** Quantity of SWCNTs eluted by 25 mM of the different solutes. The quantitative difference between NaSCN and NaCl is 6.0 ± 2.0% and that between NaSCN and NaCl/urea is 7.5 ± 0.6%

Figure 3a shows the absorption spectra, normalized at 280 nm, of the solutions collected as flow-through fractions in 1 wt% SDS solution and those collected as fractions eluted by the addition of 1 wt% DOC solution. The flow-through fraction had characteristic absorption peaks assigned to the M_11_ band; the eluted fraction had peaks assigned to the S_11_ and S_22_ bands, indicating metal/semiconductor separation, as reported in previous studies [35, 43]. Peaks remain in the S_11_ and S_22_ bands for the flow-through fraction, which are ascribed to larger-diameter semiconducting SWCNTs.

The adsorbed SWCNTs in 1 wt% SDS, i.e., semiconductor-enriched SWCNTs, were eluted by solutions containing different solutes after removing the flow-through fraction to examine their desorption effect on the SWCNTs. Figure 3b shows a representative absorption spectrum of the SWCNTs eluted with a 25 mM NaCl solution and that of the SWCNTs eluted with a subsequent 1 wt% DOC solution. The spectrum of SWCNTs eluted only with 1 wt% DOC is shown in Fig. 3b for reference and corresponds to that in Fig. 3a. Hirano et al. reported that cations have dominant effects on SWCNT desorption from hydrogel surfaces; the larger the ionic diameter is, the stronger the desorption effect is [12]. For example, Cs^+^ ions are more effective for desorption than Na^+^ ions. The 25 mM NaCl solution eluted a large quantity of SWCNTs (Fig. 3b). The SWCNTs remaining on the hydrogel surfaces in the presence of 25 mM NaCl, which were eluted by the subsequent addition of 1 wt% DOC, were mainly (6,5) tubes with an S_11_ peak at approximately 980 nm (Fig. 3b); the higher adsorbability of (6,5) tubes is due to their larger band gap [12, 37]. The same procedure was applied to the other solutes, i.e., NaSCN, urea and the NaCl/urea mixture. Figure 3c summarizes the quantity of SWCNTs eluted by these solutes, which was determined based on the absorbance values at 280 nm; note that the absorbance is insignificantly affected by light absorption of NaSCN itself under these conditions. Despite the dominant effect of sodium ions, a slight but significant difference was observed between NaCl and NaSCN (Fig. 3c), which indicates that thiocyanate ions have some elution effect, even on (6,5) tubes. In contrast, the effect of urea was comparable to that of the no additive sample. The effect of the NaCl/urea mixture was similar to that of NaCl. Therefore, NaSCN has a destabilizing effect on SDS-dispersed SWCNTs, even in the adsorption reaction. In addition, as seen for urea, there is no chaotropic effect on the stability of the SWCNTs in the adsorption reaction at low concentrations, which is consistent with the results in ATP systems. The chaotropic effect appears at higher concentrations, typically greater than 1 M [12].

### Raman Spectra of SWCNTs in Various Surfactant Solutions

Neutral salts, especially NaSCN, thermodynamically destabilize SDS-dispersed SWCNTs, which affects the association of the individual SWCNTs, the partitioning in ATP systems and the adsorption onto hydrogel surfaces. Such destabilization should depend on the nature of the surfactant. Here, alteration of the SWCNT Raman spectra by the addition of NaSCN was examined in the presence of different surfactants, namely, sodium deoxycholate (DOC) and SC. These surfactants are known to be more effective in the dispersion of SWCNTs than SDS [46–49]; in addition, they are widely used for the separation of SWCNTs [10, 50–53].

**Fig. 4:**
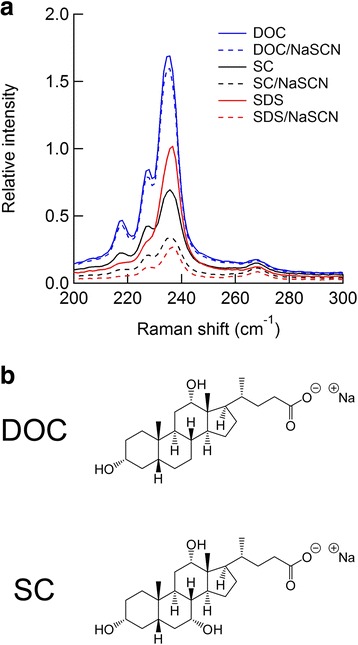
**a** Raman spectra of the SDS-dispersed SWCNTs after 2-fold dilution in 1 wt% solutions of different surfactants with or without NaSCN. The final solutions contain 0.5 wt% SDS, 0.5 wt% each surfactant and 0 or 12.5 mM NaSCN. The spectra are presented relative to the intensity at 236 cm^−1^ for the original sample, which was diluted twofold in 1 wt% SDS solution. **b** Chemical structures of DOC and SC

Figure 4a shows the Raman spectra of the SWCNTs obtained by twofold dilution of the original SDS-dispersed SWCNTs, which contained 0 or 25 mM NaSCN and 1 wt% SDS, into 1 wt% of different negatively charged surfactant solutions, i.e., DOC or SC. The Raman spectra are expressed as relative intensities to the value at 236 cm^−1^ for the control sample, which was prepared by twofold dilution of the original SWCNT dispersion without additives into a 1 wt% SDS solution. The spectral intensities were substantially increased in the presence of DOC; note that the peaks at approximately 218 and 228 cm^−1^, corresponding to (9,7) and (10,5), respectively, became more evident, which is attributed to the dense packing of DOC on the SWCNTs [14]. These intensities remained almost constant, even in the presence of NaSCN, which suggests that the DOC-dispersed SWCNTs were insignificantly destabilized by NaSCN. SC, which is a chemical similar to DOC (Fig. 4b), had a weaker effect on the intensity retention in the presence of NaSCN; namely, the destabilizing effect of NaSCN on the SWCNTs occurs even in the presence of SC, which is in concordance with the results in the ATP system containing SC and SDS (Fig. 2). Attenuation of the Raman spectra was also observed under the same conditions as in the ATP system without polymers (Additional files 1: Figure S7).

### Mechanistic Insight into the Thermodynamics of the Stability of Surfactant-Dispersed SWCNTs in the Presence of Neutral Salts

The salt-induced association of SDS-dispersed SWCNTs shown in Fig. 1 is related to the thermodynamic stability of SDS on the SWCNT surfaces; therefore, the stability is expressed using thermodynamic terms. The preferential interaction parameter, ${\left(\partial {m}_3/\partial {m}_2\right)}_{T,{\mu}_1,{\mu}_3}$, is often used to describe the thermodynamic stability of polymers, including proteins, in aqueous solutions in the presence of solutes, such as salts, where *m*_*i*_ is the molarity of component *i* and components 1, 2, and 3 are water, polymer, and solute, respectively. The preferential interaction parameter is related to the surface tension, *σ*, based on the following equation [54–56]:

1 $$ \begin{array}{l}{\left(\partial {m}_3/\partial {m}_2\right)}_{T,{\mu}_1,{\mu}_3}=-{\left(\partial {\mu}_2/\partial {m}_3\right)}_{T, P,{m}_2}/{\left(\partial {\mu}_3/\partial {m}_3\right)}_{T, P,{m}_2}\\ {}\kern8em =-{N}_{\mathrm{A}}{s}_2{\left(\partial \sigma /\partial {m}_3\right)}_{T, P,{m}_2}/{\left(\partial {\mu}_3/\partial {m}_3\right)}_{T, P,{m}_2}\end{array} $$

where *μ*_i_ is the chemical potential of component *i*, *N*_A_ is Avogadro’s number, and *s*_2_ is the surface area of one molecule of component 2. The preferential interaction parameter is applicable to surfactant micelle systems as well as polymers. Neutral salts at 0.1–0.3 M have negative preferential interaction parameter values in SDS solutions [31, 32], which means that these salts are preferentially excluded from SDS micelles. The values for NaCl and NaBr are similar at 0.3 M [32]. In addition, NaSCN has negative values, even at 0.8 M [57]. The preferential exclusion of these salts from the SDS micelles increases the surface free energy of the SDS micelles and results in thermodynamic destabilization of the micelles because the self-interaction term, ${\left(\partial {\mu}_3/\partial {m}_3\right)}_{T, P,{m}_2}$, is always positive (Eq. 1). This description is qualitatively applicable to SDS-dispersed SWCNTs. The individual state of the SDS-dispersed SWCNTs is more destabilized by these salts than the associated state since the SWCNTs in the individual state have larger solvent-exposed interfaces than those in the associated state (Eq. 1). Unfortunately, we could not obtain data for the preferential interaction parameter for NaSCN at low concentrations; however, the Raman spectra shown in Fig. 1 suggest that NaSCN more effectively destabilizes the individual state of SDS-dispersed SWCNTs.

The alteration of the thermodynamic stability of component 2 by the addition of component 3 is expressed in terms of the transfer free energy, Δ*G*_tr_, which is related to the preferential interaction parameter, ${\left(\partial {m}_3/\partial {m}_2\right)}_{T,{\mu}_1,{\mu}_3}$, as described by the following equation [56]:

2 $$ \begin{array}{l}\varDelta {G}_{\mathrm{tr}}={\displaystyle \underset{0}{\overset{m_3}{\int }}}{\left(\partial {\mu}_2/\partial {m}_3\right)}_{T, P,{m}_2}\mathrm{d}{m}_3\\ {}\kern2em =-{\displaystyle \underset{0}{\overset{m_3}{\int }}}{\left(\partial {m}_3/\partial {m}_2\right)}_{T,{\mu}_1,{\mu}_3}{\left(\partial {\mu}_3/\partial {m}_3\right)}_{T, P,{m}_2}\mathrm{d}{m}_3\end{array} $$

Neutral salts are expected to have negative preferential interaction parameters for surfactant-dispersed SWCNTs, especially for SDS-dispersed SWCNTs. On the basis of the relationship expressed in Eq. 2, the values of the transfer free energy of the SWCNTs become more positive with increasing salt concentration, leading to greater destabilization of the SWCNTs.

**Fig. 5:**
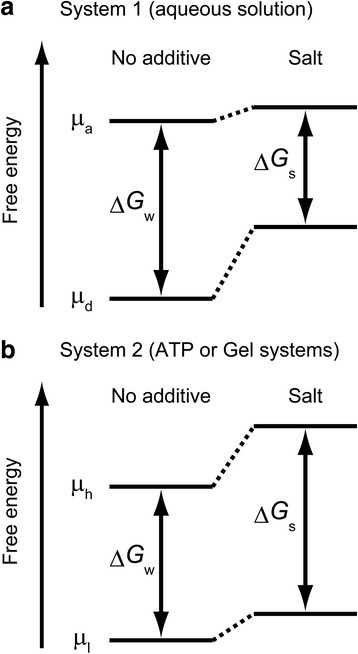
Schematic diagrams of the free energy of the surfactant-dispersed SWCNTs for the SWCNT association-governed system (system 1) and the SWCNT migration-governed system (system 2). The free energy difference (ΔΔ*G*) is calculated by subtracting the stability of SWCNTs in the no additive solution (Δ*G*_w_) from that in the salt solution (Δ*G*_s_), i.e., ΔΔ*G* = Δ*G*_s_ − Δ*G*_w_. **a** System 1: The associated SWCNTs are relatively stabilized in the presence of salts, i.e., ΔΔ*G* < 0, because the chemical potential of the individually dissociated SWCNTs (*μ*_d_) is more significantly increased by the addition of salts than that of the associated SWCNTs (*μ*_a_) owing to the difference in surface area. **b** System 2: SWCNTs in phase-*h*, i.e., the ATP top phase or the column gel phase, are relatively destabilized in the presence of salts, i.e., ΔΔ*G* > 0, because the chemical potential of the SWCNTs in phase-*h*, depicted by *μ*_h_, is more significantly increased by the addition of salts than that of the SWCNTs in phase-*l*, i.e., the ATP bottom phase or the column mobile phase, depicted by *μ*_l_. The transfer free energy (Δ*G*_tr_) is calculated by subtracting the chemical potential of SWCNTs in the no additive solution from that in the salt solution (Eq. 2) [56].

Figure 5 shows schematic diagrams of the free energy of surfactant-dispersed SWCNTs in the presence and absence of salts. When the thermodynamic equilibrium of the system is governed by the reversible association, the destabilization of SWCNTs by neutral salts shifts the equilibrium of the reaction toward the associated state (Fig. 5a). Accordingly, the SWCNT association becomes more pronounced with increasing salt concentration (Fig. 1). In contrast, when the thermodynamic equilibrium is dominated by partitioning into a biphasic system or by adsorption onto hydrogel surfaces, the destabilization by the salts causes migration of SWCNTs from the high-concentration-salt phase (phase-*h*) to the low-concentration-salt phase (phase-*l*) (Fig. 5b). Taken together, neutral salts alter the colloidal stability of the surfactant-dispersed SWCNTs through the thermodynamic destabilization caused by their preferential exclusion from surfactants on the SWCNT surfaces. An important future goal is to identify the preferential interaction parameter of NaSCN for the surfactant-dispersed SWCNTs by subtracting the contribution of the coexisting surfactant micelles for quantitative analyses of the transfer free energy and to determine the molecular mechanism of the preferential interaction.

## Conclusions

Neutral salts at concentrations less than 25 mM affected the colloidal stability of surfactant-dispersed SWCNTs, including the association, partitioning, and adsorption propensities. NaSCN had a prominent destabilizing effect on the SWCNTs. The alteration of the thermodynamic stability of the SWCNTs is attributable to the preferential exclusion of salts from the surfactant assemblies on the SWCNTs. Although NaSCN was previously proposed to behave as a stabilizing agent for SWCNTs owing to its potential chaotropic effect, the present results suggest that NaSCN acts as a destabilizing agent at concentrations of at least 25 mM. These findings provide a basis for understanding the colloidal behavior of surfactant-dispersed SWCNTs in electrolyte solutions and for developing the separation chemistry of SWCNTs.

## Additional file

Additional file 1: Experimental Results. (DOCX 636 kb)

## Abbreviations

ATP: Aqueous two-phase
DOC: Sodium deoxycholate
HiPco: High-pressure catalytic CO
PEG: Polyethylene glycol
PL: Photoluminescence
RBM: Radial breathing mode
SC: Sodium cholate
SDS: Sodium dodecyl sulfate
SWCNT: Single-wall carbon nanotube
