# Supplementary material for: Destabilization of Surfactant-Dispersed Carbon Nanotubes by Anions
Source: Nanoscale Res Lett. 2017 Jan 31;12:81. doi: 10.1186/s11671-017-1850-1 (PMC5280815; doi:10.1186/s11671-017-1850-1)
Supplement: Additional file 1: — Experimental Results. (DOCX 636 kb) [file 11671_2017_1850_MOESM1_ESM.docx]

**Additional file 1**

Destabilization of Surfactant-Dispersed Carbon Nanotubes by Anions

Atsushi Hirano,^a,*^ Weilu Gao,^b^ Xiaowei He,^b^ Junichiro Kono^b,c,d^

^a^Nanomaterials Research Institute, National Institute of Advanced Industrial Science and Technology (AIST), Tsukuba, Ibaraki 305-8565, Japan

^b^Department of Electrical and Computer Engineering, Rice University, Houston, Texas 77005, USA

^c^Department of Physics and Astronomy, Rice University, Houston, Texas 77005, USA

^d^Department of Materials Science and NanoEngineering, Rice University, Houston, Texas 77005, USA

* To whom correspondence should be addressed: Nanomaterials Research Institute, National Institute of Advanced Industrial Science and Technology, Tsukuba, Ibaraki 305-8565, Japan. Tel.: +81-29-849-1064, Fax: +81-29-861-2786, E-mail: hirano-a@aist.go.jp

The absorption spectra of the SDS-dispersed SWCNTs were measured in the presence and absence of various solutes, i.e., HCl and K_2_IrCl_6_, which have been reported to oxidize SWCNTs by following the O_2_/H_2_O redox couple (1230 mV vs. NHE) and the Ir(IV)/Ir(III) redox couple (867 mV vs. NHE). The spectral intensities in the S_11_ band decreased in the presence of 0.5 mM HCl, where the addition of HCl resulted in a final solution pH of 4, whereas the spectral intensities disappeared in the presence of 0.5 mM K_2_IrCl_6_ (Fig. S1). Based on these results, the effect, if any, of the neutral salts used in the present study on the redox chemistry of SWCNTs is negligible compared to the conventional oxidation reagents at less than 25 mM. Importantly, Xu et al. reported, on the basis of absorption spectroscopy, that NaSCN itself does not to react with SWCNTs dispersed by single-stranded DNA in the absence of H_2_O_2_, which is consistent with the present results.


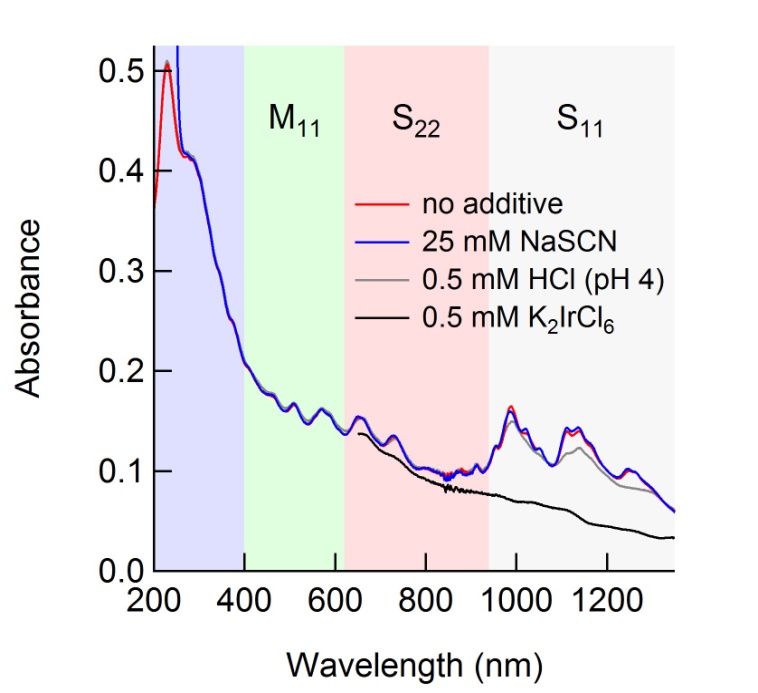


**Fig. S1.** Absorption spectra of the SWCNTs in the presence and absence of different oxidants.


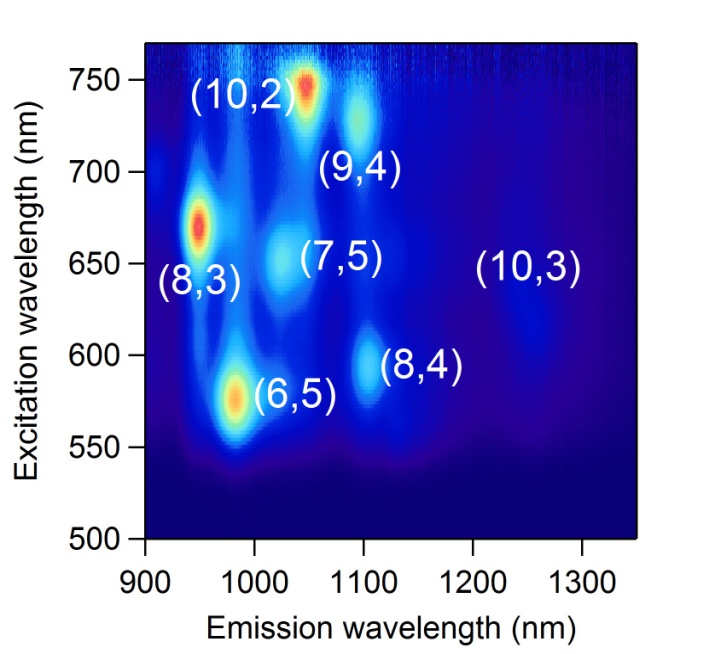


**Fig. S2.** Representative photoluminescence excitation spectra of SWCNTs dispersed by 1 wt % SDS.

**
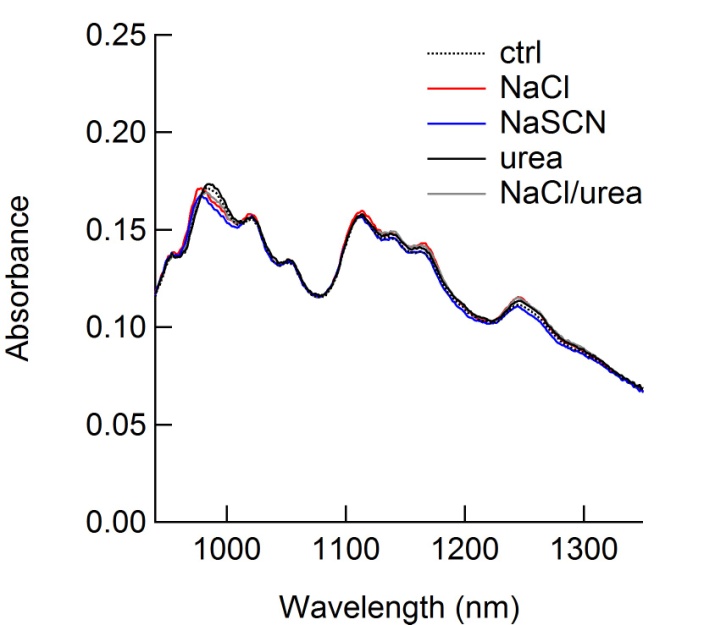
**

**Fig. S3.** Representative absorption spectra of SWCNTs in the S_11_ band with 25 mM solutes in 1 wt % SDS solution. The spectra were measured using a UV–vis–NIR spectrophotometer (UV-3101PC, Shimadzu).

**Thermodynamic Measurements for Phenyl Group Solubilization Using Amino Acids**

The chaotropic effects of the solutes on the aromatic surfaces of the SWCNTs were examined by estimating their thermodynamic stabilization effect on two amino acids, i.e., phenylalanine and alanine (Fig. S4). One useful approach to quantifying the stabilization effects on amino acids is to determine the transfer free energy, which was developed by Nozaki and Tanford (Nozaki, Y; Tanford, C, *J. Biol. Chem.* **1963**, *238*, 4074–4081). In the present study, the thermodynamic stabilization effects of NaCl, NaSCN and urea on the aromatic group ($\Delta G_{\mathrm{tr}}^{\text{Ar}}$) were estimated by subtracting the transfer free energy of alanine ($\Delta G_{\mathrm{tr}}^{\text{Ala}}$) from that of phenylalanine $(\Delta G_{\mathrm{tr}}^{\text{Phe}}$). The transfer free energy is defined below.


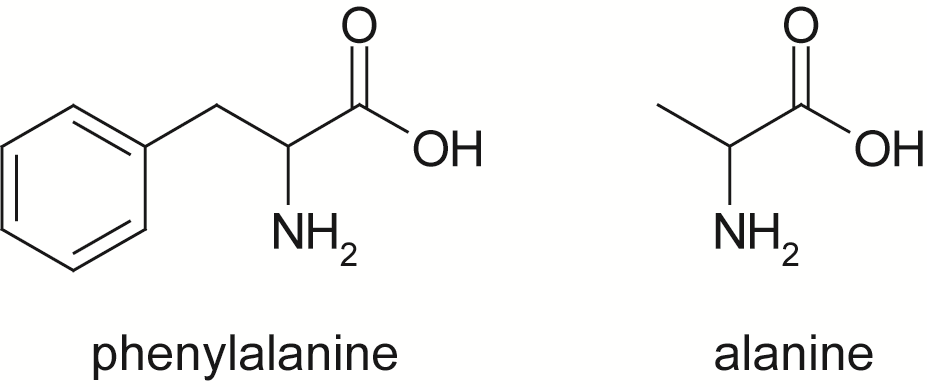


**Fig. S4.** Chemical structures of phenylalanine and alanine.

At equilibrium, the chemical potentials of the amino acids in the water ($\mu_{w}$) are equal to those in solutions containing the solutes ($\mu_{s}$); the transfer free energy of the amino acids from water to solutions containing the solutes (additive solutions) is expressed by the following equation:

${\Delta G}_{\mathrm{tr}}=\mu_{s}^{0}-\mu_{w}^{0}=-RT\ln\left( S_{s}/S_{w} \right)\approx-RT\ln\left( n_{a,s}/n_{a,w} \right)$ (1)

in which

$\left\{ \begin{aligned} \mu_{w}^{0}=\mu_{w}-RT\ln S_{w} \\ \mu_{s}^{0}=\mu_{s}-RT\ln S_{s} \end{aligned} \right.$ (2)

$\left\{ \begin{aligned} S_{w}=n_{a,w}/(n_{a,w}+n_{w,w}) \\ S_{s}=n_{a,s}/(n_{a,s}+n_{w,s}+n_{s,s}) \end{aligned} \right.$ (3)

In these equations, the activity coefficients of the amino acids were assumed to be unity. $\mu_{w}^{0}$ and $\mu_{s}^{0}$ are the standard chemical potentials of the amino acids in water and in the additive solutions, respectively. $S_{w}$ and $S_{s}$ represent the solubility of the amino acids, which correspond to the mole fractions of amino acids in water and in the additive solutions at solubility equilibrium, respectively. Therefore, $n_{i,w}$ and $n_{i,s}$ are the molarities of component *i* in water and in the additive solutions at equilibrium, respectively, where the subscripts *a*, *w*, and *s* corresponding to *i* denote the amino acids, water, and added solutes, respectively. In these equations, *R* and *T* represent the gas constant and absolute temperature, respectively.

The solubilities of the amino acids were determined at 25°C. Excess quantities of amino acids were suspended in the different solutions. The suspensions were brought to solubility equilibrium at 25°C by incubation for at least 16 hours. The suspensions were then gently centrifuged at 25°C to obtain the saturated supernatants. The concentrations of the amino acids in the supernatants were determined by high-performance liquid chromatography with a C18 column and an absorption spectrometer; phenylalanine and alanine were detected at 257 nm and 200 nm, respectively. A 10 mM sodium phosphate buffer solution at pH 7 for phenylalanine and a 50 mM acetate solution for alanine were used as the mobile phases at a flow rate of 1.0 ml/min.

The transfer free energy for phenylalanine and alanine are listed in Table S1. NaCl destabilized phenylalanine, whereas NaSCN and urea stabilized it; note that NaSCN was more effective than urea. In contrast, NaCl did not affect the stability of alanine. NaSCN stabilized alanine, while urea destabilized it. To extract the contribution of these solutes to the stability of the aromatic group ($\Delta G_{\mathrm{tr}}^{\text{Ar}}$), the transfer free energy of alanine ($\Delta G_{\mathrm{tr}}^{\text{Ala}}$) was subtracted from that of phenylalanine $(\Delta G_{\mathrm{tr}}^{\text{Phe}}$) for each solute. The results indicate that NaCl destabilized the aromatic group, whereas NaSCN and urea stabilized it to a similar extent. Based on these results, NaSCN and urea have similar thermodynamic stabilizing effects on the aromatic surfaces of SWCNTs.

**Table S1.** Transfer free energy of the amino acids from water to different solutions at 1 M.*^a^*

| solute | Transfer free energy (J/mol) | | |
| --- | --- | --- | --- |
|  | $\Delta G_{\mathrm{tr}}^{\text{Phe}}$ | $\Delta G_{\mathrm{tr}}^{\text{Ala}}$ | $\Delta G_{\mathrm{tr}}^{\text{Ar}}$ |
| NaCl | 329±8 | −4±32 | 333±33 |
| NaSCN | −419±9 | −64±25 | −355±26 |
| Urea | −281±10 | 78±25 | −359±27 |

*^a^* $\Delta G_{\mathrm{tr}}^{\text{Ar}}=\Delta G_{\mathrm{tr}}^{\text{Phe}}-\Delta G_{\mathrm{tr}}^{\text{Ala}}$

**
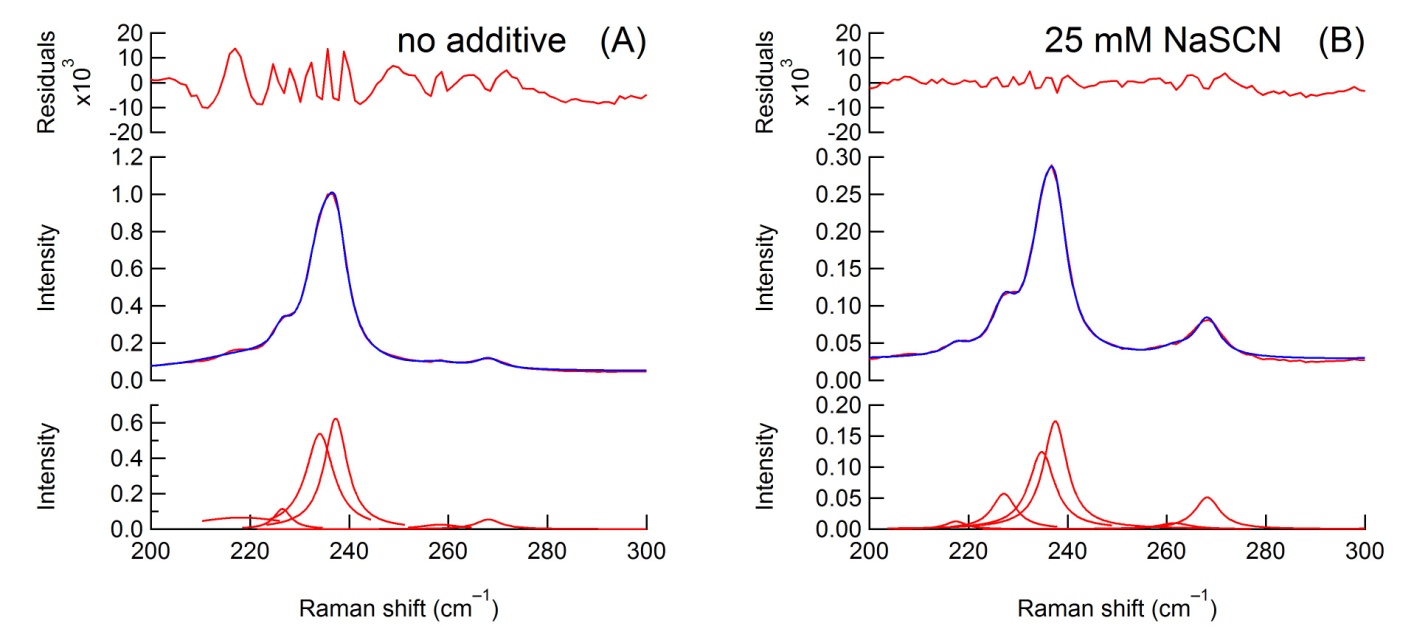
**

**Fig. S5.** Lorentzian decomposition of the Raman spectra. Residuals of the fitting are shown in top panels. The raw data and the fitting curves are depicted by red and blue lines, respectively (middle panels), and the decomposed spectra (bottom panels) are presented for no additive (A) and 25 mM NaSCN (B). The peak intensities at 268 cm^−1^ were 0.054 for no additive and 0.051 for 25 mM NaSCN.

**
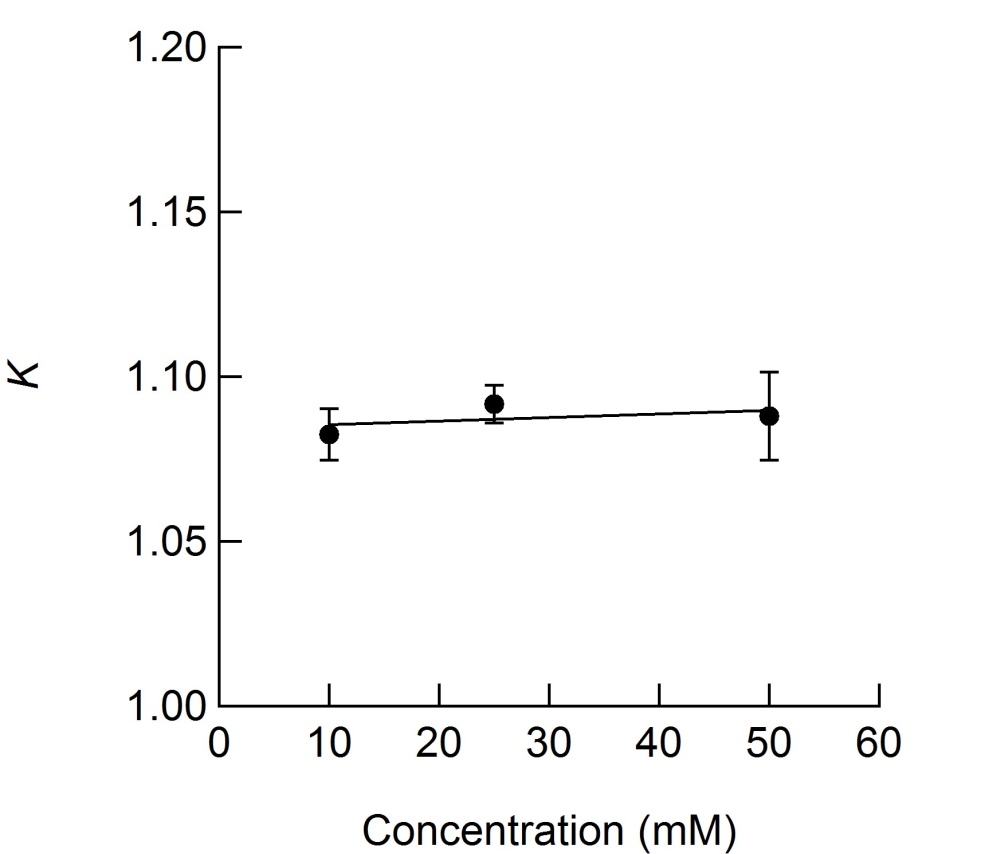
**

**Fig. S6.** Partition coefficients of NaSCN, *K* = *C*_top_/*C*_bottom_, at 10–50 mM in the ATP system. The equilibration of the system was performed by mixing a solution containing 6 wt % PEG (polyethylene glycol 6,000, Alfa Aesar), 6 wt % dextran (Dextran 70, Tokyo Chemical Industry, Japan), 0.4 wt % SDS (Wako Pure Chemical Industries, Japan) and 0.9 wt % SC (Tokyo Chemical Industry, Japan) at 25°C for more than 12 hours. The concentration of NaSCN in each phase was determined by measuring the absorbance at 220 nm after 200-fold dilution; the marginal background absorbance originating from the polymers and the surfactants was subtracted from the value of each sample.


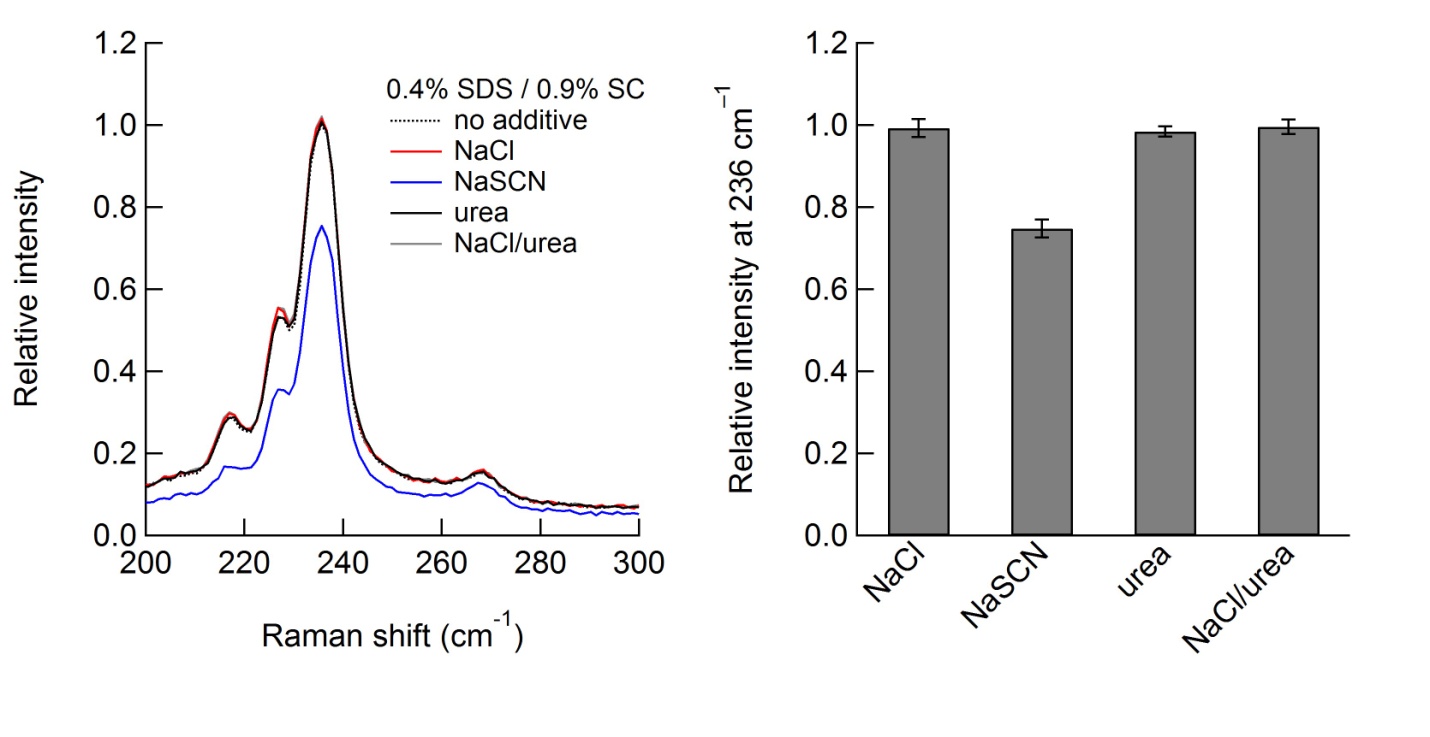


**Fig. S7.** Raman spectra of the SWCNTs in 0.4 wt % SDS and 0.9 wt % SC solutions with or without solutes. The spectra are presented relative to the intensity at 236 cm^−1^ for the sample in the absence of solutes. Only NaSCN showed significant attenuation of the spectral intensity. The pH difference among these samples was less than 0.2.
